# Treatment of Bilateral Empyema of Maxillary Sinuses

**Published:** 1904-05-15

**Authors:** George Zederbaum

**Affiliations:** Charlotte, Mich.


### TREATMENT OF BILATERAL EMPYEMA OF MAXILLARY SINUSES

BY GEORGE ZEDERBAUM, D.D.S., CHARLOTTE, MICH.

A young man, aged twenty, presented himself for examination. He complained of pain in the regions immediately above both first upper molars. There was noticeable considerable swelling, heat, and upon digital pressure pain was produced. The two first molars mentioned, as well as many other teeth, were badly decayed. The mouth in general was very unclean and malodorous. Temperature 102 degrees. Patient's history bad. Catarrhal inflammation of Schneiderian membrane, with its constant acrid discharges still present. Throat diphtheritic (his mother had lost five children within two weeks from the last named disease.) Patient weighed 115 pounds, although he was a tall fellow, and should have weighed about 160. Altogether in a very unfavorable, emaciated and neurasthenic condition.

Procedure: The two upper first molars being beyond repair were extracted, and on their removal a quantity of pus came from each socket. Upon examining the roots of the extracted teeth, I noticed the apical foramina of buccal roots considerably enlarged. Each time the patient lowered his head to the cuspidor a quantity of very foul fluid was discharged from each nostril. As soon as the blood ceased to flow, each antrum was easily reached with a silver probe, and direction of sinus determined in both cases. The openings were enlarged sufficiently for the introduction of an abscess syringe point, and both sinuses were thoroughly washed out with a fifty percent solution of glycothymoline (Kress). Then the wounds were dressed with a five-percent moist iodoform gauze. At same sitting all the remaining teeth were thoroughly scaled and cleansed. The patient was then dismissed, with orders to take one ounce of sulphate of magnesia, and to snuff twenty-five percent solution of glycothymoline half hourly.

The young man returned the following day, with temperature of 104 degrees, and the right side considerably worse than the left. More swelling, amaurosis of right eye, with considerable bulging. Old dressings were removed and found dripping with pus. A quantity of necrosed bone was removed from both sinuses; then all the inner walls curetted and again washed out with fifty percent glycothymoline, and packed as before. He was sent to bed with instructions for quinine sulphate, three grains, three times a day, and frequent rinsing out of nose with twenty-five percent solution glycothymoline. His temperature was 101 degrees before he left the office.

This treatment was continued for two weeks, dressings being changed every other day and packed more loosely each time. At the end of that time the discharge was not noticeable and wounds almost entirely healed. A warm solution of cassia water was recommended as a permanent mouth wash. In addition to this the patient was then put on elixir of quinine, iron and strychnia—thirty drops in a little water, with meals. As a result, the patient gained ten pounds in three weeks, and was discharged.

I attribute the early resolution of this case to drugs used and the strictly aseptic conditions under which all the work was done. The young man has had other work done by me since, and now that five months have elapsed since the antrum trouble, and no signs of recurrence are present, I regard the case as completely cured.

The pleasantest feature of this case, however, was that the young man, although poor, paid me twice the amount I charged.—*Review.*

